# Effective high-dose methotrexate toxicity reversal using fixed-dose glucarpidase in obese patients: a case series

**DOI:** 10.1186/s13256-025-05774-2

**Published:** 2026-03-06

**Authors:** John Grofvert, Brett Van Rossum, Erin Pettijohn, Mohammad Mobayed, Devon Stonerock

**Affiliations:** 1 Department of Pharmacy, Corewell Health Grand Rapids Hospital, Grand Rapids, MI USA; 2 The Cancer and Hematology Centers, Grand Rapids, MI USA; 3 Department of Medical Oncology, Corewell Health Beaumont Troy Hospital, Troy, MI USA

**Keywords:** Glucarpidase, Methotrexate, Nephrotoxicity, Delayed clearance, Case report

## Abstract

**Background:**

This case report and literature review present two instances of delayed methotrexate clearance in individuals who were successfully managed using fixed dosing versus weight-based dosing of glucarpidase. These cases, along with existing literature, support the use of fixed-dose glucarpidase as an alternative to weight-based dosing for delayed methotrexate clearance.

**Case presentation:**

Patient 1: a 26-year-old obese African American male with newly diagnosed osteosarcoma of the right distal fibula underwent his first cycle of neoadjuvant chemotherapy with MAP. Following the administration of doxorubicin and cisplatin, the patient developed acute kidney injury, with increased serum creatinine. He was admitted for high-dose methotrexate administration. Leucovorin rescue began 24 hours after high-dose methotrexate infusion. Twenty-five hours post-infusion, the patient had an elevated serum creatinine and methotrexate levels. A fixed dose of glucarpidase (2000 units) was administered, resulting in rapidly declining methotrexate levels and patient discharge. Patient 2: a 77-year-old obese white male with primary central nervous system lymphoma was admitted for cycle 3 of high-dose methotrexate and rituximab as first-line treatment. His pretreatment comorbidities included chronic kidney disease, coronary artery disease, hypertension, pulmonary hypertension, and chronic obstructive pulmonary disease. Following rituximab and high-dose methotrexate administration, nephrology was consulted owing to elevated creatinine. Leucovorin rescue began 24 hours after high-dose methotrexate treatment. On day 2, creatinine remained elevated with a post-dose methotrexate level indicating potential delayed clearance. A fixed dose of glucarpidase (2000 units) was administered, resulting in rapidly declining methotrexate levels and patient discharge. Cycle 4 was performed without complications, but cycle 5 required a fixed dose of glucarpidase owing to potential delayed clearance, resulting in rapidly declining methotrexate levels and patient discharge.

**Conclusion:**

In patients receiving high-dose methotrexate therapy requiring glucarpidase therapy, transitioning to a fixed dosing approach has significant cost-saving implications for institutions.

**Supplementary Information:**

The online version contains supplementary material available at 10.1186/s13256-025-05774-2.

## Background

Methotrexate (MTX) is an essential chemotherapeutic agent used to treat various malignancies, including osteosarcoma, leukemia, and lymphoma. Its primary mechanism of action is the inhibition of dihydrofolate reductase, an enzyme crucial for folate metabolism; however, several other mechanisms exert its effects [1]. In the treatment of osteosarcoma and central nervous system lymphoma (CNSL), high-dose methotrexate (HDMTX), which is traditionally defined as ≥ 500 mg/m^2^, is frequently used, and can lead to significant toxicity if not managed appropriately [2].

Leucovorin, a reduced folate, is administered as part of the methotrexate regimen to rescue nonmalignant cells from the toxic effects of methotrexate by bypassing the blockade in folate metabolism [3]. Delayed clearance of methotrexate, especially in the setting of renal dysfunction, can result in severe toxicities owing to effects on nonmalignant cells, including nephrotoxicity, mucositis, and myelosuppression, which can be fatal if not treated promptly. Cisplatin, another antineoplastic agent often used in combination with methotrexate (for example, in MAP [high-dose methotrexate, doxorubicin, and cisplatin] therapy for osteosarcoma), can exacerbate nephrotoxicity and further impair methotrexate clearance [3–5].

Glucarpidase, a recombinant enzyme derived from *Escherichia coli*, effectively reduces methotrexate-induced toxicity in individuals with delayed methotrexate clearance, independent of body mass index (BMI) [6]. Administration results in a rapid reduction in serum methotrexate levels, eliminating the need for hemodialysis in some cases [7]. While effective, glucarpidase is listed at a high cost in the USA, commonly exceeding US$100,000 per dose for adult patients, necessitating investigation into alternative dosing strategies. The package insert recommended dosing of glucarpidase is 50 units/kg as a single dose; however, this was based on *in vitro* data, and early dose-finding studies were not performed [8]. Glucarpidase has a rapid onset of action and is characterized by a small volume of distribution (68 mL/kg), approximating the average human intravascular volume of distribution. The pharmacokinetic profile of glucarpidase suggests that its efficacy is largely confined to the intravascular space, making body weight and adiposity less relevant to dosing. This supports the rationale for fixed-dose administration, as the enzyme’s activity does not scale proportionally with body mass. Moreover, saturation kinetics observed in clinical settings indicate that once enough enzyme is present to catalyze methotrexate breakdown, additional dosing yields diminishing returns [8, 9]. Consequently, fixed dosing not only simplifies administration but also offers a cost-effective alternative without compromising therapeutic efficacy, particularly in patients with renal impairment regardless of their BMI. Herein, we present two successful cases of fixed dose glucarpidase administration.

## Case presentation 1

A 26-year-old African American male with obesity (BMI of 33 kg/m^2^), limited medical history, and a family history of brain cancer and an unspecified malignancy on the maternal side, was recently diagnosed with osteosarcoma of the right distal fibula. One month post-diagnosis, the patient initiated neoadjuvant chemotherapy with MAP. Following doxorubicin (37.5 mg/m^2^) and cisplatin (60 mg/m^2^) administration on day 0, the patient developed acute kidney injury (AKI), peaking at a serum creatinine (SCr) level of 2.16 mg/dL on day 9 (baseline: 0.8–1 mg/dL). On day 23, the patient was admitted for high-dose methotrexate (HDMTX) administration, with SCr reduced to 1.42 mg/dL. Liver function tests were within normal limits at baseline. No medications known to delay methotrexate clearance were administered within 48 hours prior to high-dose methotrexate (HDMTX) treatment. Owing to residual AKI, the HDMTX dose was halved to 6000 mg/m^2^ and administered over 4 hours [10]. Hydration with sodium bicarbonate infusion was initiated to maintain a urine pH > 7. Leucovorin rescue began 24 hours post-HDMTX infusion at 50 mg every 6 hours.

**Fig. 1:**
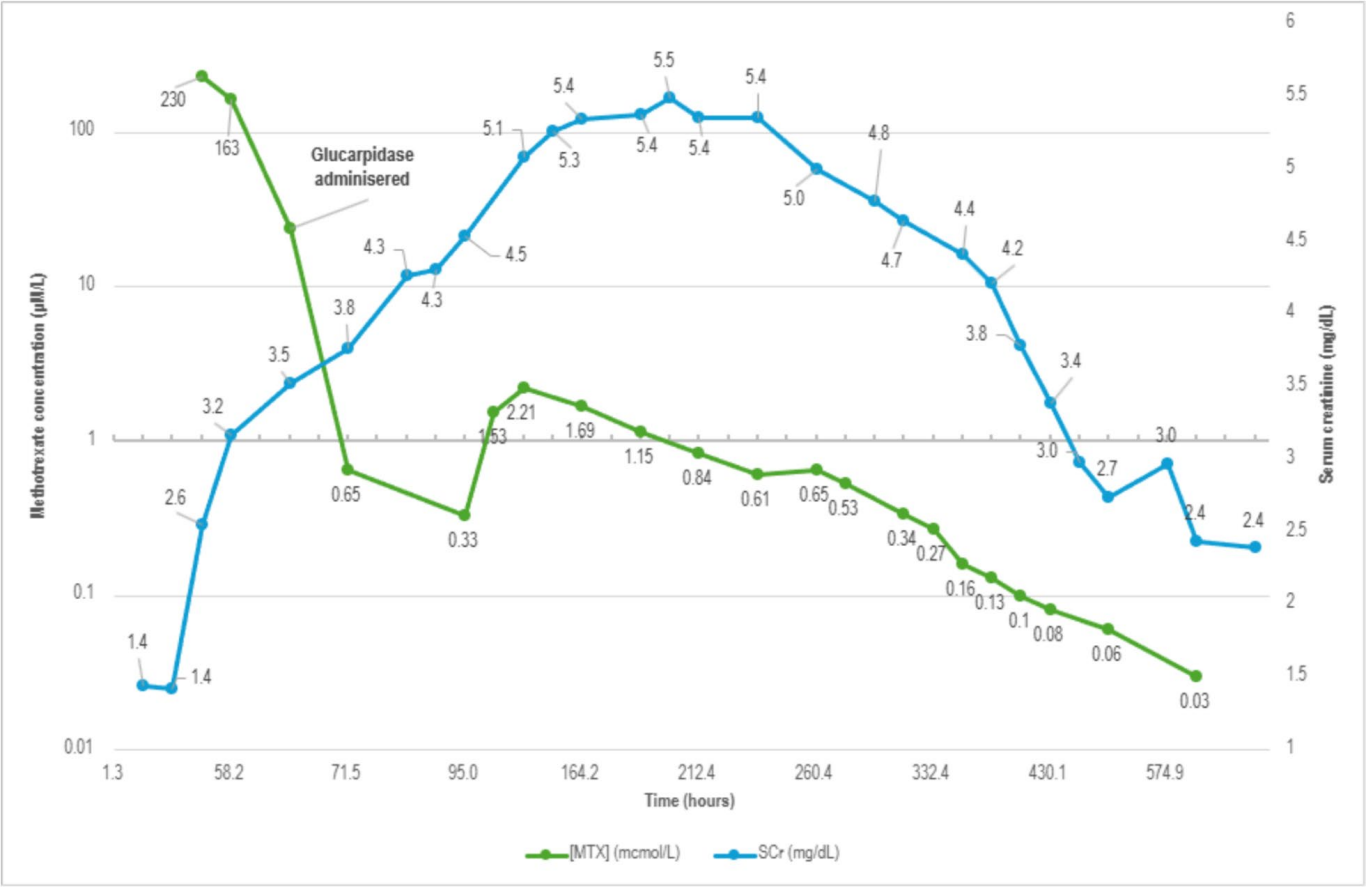
Methotrexate serum concentrations and serum creatinine levels during treatment for Patient 1

An Initial elevated SCr (2.55 mg/dL) and methotrexate level (229.94 µmol/L) drawn 25-hours post-MTX infusion indicated nephrotoxicity and delayed clearance. The patient’s predicted clearance was modeled on MTXPK.org (Supplementary Fig. 1) by a clinical pharmacy specialist and demonstrated that this level was > 2 standard deviations above the population average [11]. The clinical pharmacist and attending hematologist discussed the response to this level. The leucovorin dose was increased, and a fixed dose of glucarpidase (2000 units) was administered on day 24. Trends in serum creatine and MTX levels, as measured by liquid chromatography–mass spectrometry (LC–MS), are depicted in Fig. 1. The first post-glucarpidase methotrexate levels rapidly declined to 0.65 µmol/L. Methotrexate levels continued to decline, with SCr improving to 2.74 mg/dL at discharge. The patient was discharged on day 40 when methotrexate levels fell below 0.1 µmol/L. SCr returned to baseline (1–1.2 mg/dL) over several months.

This case demonstrates rapid reductions in serum methotrexate (MTX) levels following a fixed dose of glucarpidase, as opposed to the weight-based dosing of 50 units/kg. Five days after HDMTX administration, an anticipated rebound in the methotrexate level to 1.53 µmol/L was observed, maintaining a > 95% reduction from original levels. Glucarpidase serum activity levels observe a half-life of 5.6 hours, which may be prolonged with impaired renal function, thus activity is expected to diminish after 36–72 hours [8, 12, 13]. This results from the redistribution of methotrexate from peripheral tissues to the intravascular space or via clearance by anti-glucarpidase antibodies, as described previously [13, 14]. The rebound can also occur earlier owing to the formation of anti-glucarpidase antibodies, but this has only been observed after receipt of multiple prior glucarpidase doses, making this mechanism unlikely [15]. In all reports to date, similar rebounds in methotrexate levels were observed and similarly remained > 95% lower than pre-glucarpidase levels [7, 16–19]. We hypothesize that patient obesity increased the extravascular methotrexate concentrations in this case, resulting in redistribution after glucarpidase activity diminished. Leucovorin was held for a period of 2-hours before and 2-hours after glucarpidase administration according to recommendations [13].

## Case presentation 2

A 77-year-old white male with obesity (BMI 36 kg/m^2^) and recently diagnosed primary central nervous system lymphoma was admitted for cycle 3 of high-dose methotrexate and rituximab (MR) as first-line treatment within 2 months of diagnosis. The patient had no reported family history of malignancy. Other pretreatment comorbidities included chronic kidney disease (baseline SCr 1.6–1.9 mg/dL), coronary artery disease, hypertension, pulmonary hypertension, and chronic obstructive pulmonary disease. Liver function tests were within normal limits at baseline. Pertinent medications included sacubatril/valsartan, metoprolol succinate, and daily bumetanide. Pantoprazole, which may impair methotrexate clearance, was discontinued the day prior to treatment [20]. No other interacting medications (for example, penicillins, nonsteroidal anti-inflammatory drugs) were administered within 48 hours before therapy initiation. Rituximab (375 mg/m^2^) was administered on day 1, followed by HDMTX (2000 mg/m^2^) over a 4-hour bolus infusion. Nephrology was consulted owing to elevated creatinine (2.06 mg/dL) on day 1. Leucovorin rescue began 24 hours post-HDMTX at 50 mg every 6 hours. SCr peaked at 2.84 mg/dL on day 2. A 24-hour post-dose MTX level was 72 µmol/L, indicating potential delayed clearance. MTX levels at this hospital were determined via an immunoassay. A 48-hour post-dose MTX level was 11.25 µmol/L. On day 4, the MTX level was 5.79 µmol/L, and the SCr doubled, so a fixed dose of glucarpidase (1000 units) was administered after discussion between the attending physician and clinical pharmacy specialist. A send-out LC–MS MTX level on day 5 was < 0.05 µmol/L. The send-out laboratory results took approximately 1 day. Immunoassay-based levels, which continued during this period, did not reflect true MTX concentrations as described previously, owing to the inability to distinguish metabolites [13]. This underscores the importance of having workflows for post-glucarpidase MTX level monitoring and distinguishing institutional laboratory methodologies. The patient was discharged on day 8 after MTX administration.

For the subsequent cycle (cycle 4) of MR, the patient’s methotrexate dose was reduced to 1500 mg/m^2^ owing to prior tolerance. SCr was 2.23 mg/dL on the day of MTX treatment. This cycle was without complications, and MTX cleared to < 0.1 µmol/L by day 8. For cycle 5, with a baseline creatinine of 1.79 mg/dL, an 18-hour post-MTX level was 64.99 µmol/L, indicating recurrent delayed clearance. Owing to a ≥ 30% increase in SCr and clinical decline (hypoxia, altered mental status, and oliguria), a fixed dose of 2000 units of glucarpidase was administered, and leucovorin dosing was increased. Post-glucarpidase levels declined but were not interpretable owing to use of immunoassays at this center. A post-glucarpidase LC–MS MTX level collected on day 4 showed < 0.05 µmol/L on day 6, indicating adequate reversal and clearance

## Discussion and conclusion

This case series underscores the importance of individualized treatment strategies when managing high-dose methotrexate toxicity, particularly in patients with renal impairment. The two patients presented—one a young African American male and the other an elderly white male—highlight how factors such as polypharmacy, age, ethnicity, and socioeconomic context can influence clinical outcomes. In older adults, polypharmacy is common and can complicate methotrexate clearance owing to potential drug interactions and cumulative renal burden. The elderly patient in this series had multiple comorbidities managed with various medications, which may have contributed to delayed methotrexate elimination [21]. Age-related decline in renal function further exacerbates this risk, even in the absence of overt kidney disease [21]. Ethnicity may also play a role, as genetic polymorphisms affecting renal transporters and folate metabolism can vary across populations and influence methotrexate pharmacokinetics [6]. Although pharmacogenomic testing was not performed, the successful use of fixed-dose glucarpidase in an African American patient supports its applicability across diverse groups. Socioeconomic factors, including access to specialized laboratory testing (for example, LC–MS for methotrexate levels), availability of glucarpidase, and hospitalization costs, are also critical. Fixed-dose glucarpidase simplifies administration and may reduce overall healthcare expenditures, making it a practical and equitable option in resource-limited settings. These cases underscore the importance of individualized dosing strategies for high-dose methotrexate therapy, particularly in patients with renal dysfunction. Reduced methotrexate dosing owing to pre-existing nephrotoxicity and fixed-dose glucarpidase administration effectively reduced methotrexate levels without compromising patient safety. Methotrexate levels continued to decline post-glucarpidase, suggesting that fixed dosing is a viable alternative to weight-based dosing, potentially reducing overall healthcare costs.

Multiple publications have highlighted the potential of reduced glucarpidase dosing strategies, as outlined in Table 1. For instance, a retrospective analysis by Heuschkel *et al*. demonstrated that patients receiving reduced glucarpidase doses (20–30 units/kg) still achieved clinically significant reductions in methotrexate levels, with comparable outcomes to weight-based dosing [16]. Other reports have suggested that fixed dosing of approximately 2000 units is effective, regardless of patient weight, which could reduce healthcare costs substantially without compromising patient outcomes. For example, a study by Schaff *et al*. observed that patients treated with fixed glucarpidase doses of 2000 units achieved similar reductions in methotrexate levels compared with weight-based dosing [15]. This finding is consistent with other retrospective analyses that advocate for cost-effective strategies, particularly in the context of methotrexate-induced nephrotoxicity, where prolonged hospitalizations and additional supportive care (for example, hemodialysis) can further increase healthcare costs.

**Table 1:** Current publications reporting the efficacy of fixed-dose glucarpidase administration as an alternative to weight-based dosing

	Patients included	Glucarpidase dose, median (range)	Time from MTX to glucarpidase administration, median or mean (range)	Age, mean (range)	Peak serum creatinine, mean or median (range)	Methotrexate dose, mean (range)	Days to methotrexate clearance^*^, mean (range)
Truong *et al*.	5	1000 units (6.4–14.5 units/kg)	53 hours (51–59)	64 (38–74)	3.14 mg/dL (0.67–7.71)	8.2 g (2.7–20)	17 days (12–24)
Widemann *et al*.	1	1000 units (7.7 units/kg)	6 days	59	4.54 mg/dL	4.1 g	11 days
Heuschkel *et al*.	7	2000–3000 units (17–32 units/kg)	56 hours (42–62)	55 (19–71)	3.42 mg/dL (2.19–7.02)	8.1 g (3–25.2)	18 days (two patients expired)
Scott *et al*.	26	50 units/kg (13–90 units/kg)	44.5 hours (26–95)	11.7 (4–20)	1.75 mg/dL (0.7–4.8)	7.4 g/m^2^ (2.5–12.4)	NR
Schaff *et al*.	8	1000–2000 units	24 hours per protocol	70 (57–78)	NA	3–6 mg/m^2^	NR

*NR* Not Reached

The gradual improvement in renal function observed in these patient cases aligns with previous studies, indicating that glucarpidase not only reduces methotrexate levels but may also expedite recovery from methotrexate-associated nephrotoxicity. Early glucarpidase administration likely mitigated further renal damage.

These cases highlight the potential benefits of fixed-dose glucarpidase in patients with delayed methotrexate clearance owing to renal impairment. A fixed dose, even at a fraction of the weight-based dose, was sufficient to rapidly reduce methotrexate levels. Given the high cost of glucarpidase, this approach may offer a cost-effective and safe alternative to traditional dosing. An institutional algorithm (Supplementary Fig. 2) has been developed at our institution to guide a safe approach to fixed dosing, with estimated annual cost savings exceeding US$500,000. A key safety measure in implementing this algorithm was to ensure that all sites have access to LC–MS for MTX level monitoring post-glucarpidase. A limitation of current evidence is the lack of randomization to a fixed versus weight-based strategy. Given the high cost of the medication and infrequent need for use, these trials are unlikely to be performed. Further research is needed to confirm optimal dosing strategies, but current evidence supports fixed or reduced dosing as an effective and safe strategy.

## Supplementary Information

Additional file 1. Methotrexate kinetic modeling for case presentation 1 by MTXPK.org.

Additional file 2. Institutional algorithm for fixed-dose glucarpidase administration.

## Data Availability

Not applicable.

## Abbreviations

MTX: Methotrexate
CNSL: Central nervous system lymphomas
HDMTX: High-dose methotrexate
MAP: High-dose methotrexate, doxorubicin, and cisplatin
BMI: Body mass index
AKI: Acute kidney injury
SCr: Serum creatinine
LC–MS: Liquid chromatography–mass spectrometry
MR: High-dose methotrexate and rituximab
