# Supplementary figures and images for: Effective high-dose methotrexate toxicity reversal using fixed-dose glucarpidase in obese patients: a case series
Source: J Med Case Rep. 2026 Mar 6;20:193. doi: 10.1186/s13256-025-05774-2 (PMC13081509; doi:10.1186/s13256-025-05774-2)

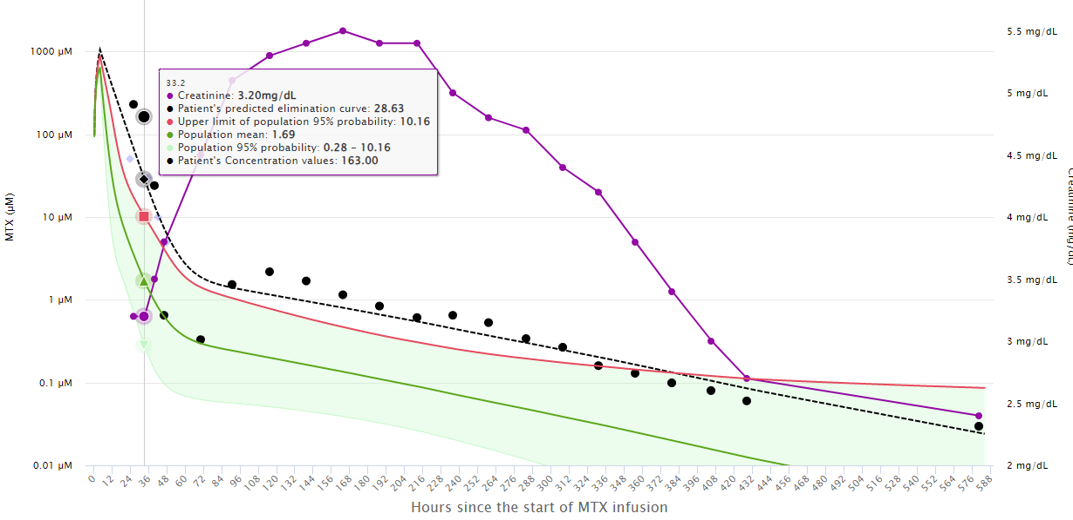

Supplement: Supplementary file 1 — Additional file 1. Methotrexate kinetic modeling for case presentation 1 by MTXPK.org. [file 13256_2025_5774_MOESM1_ESM.docx]

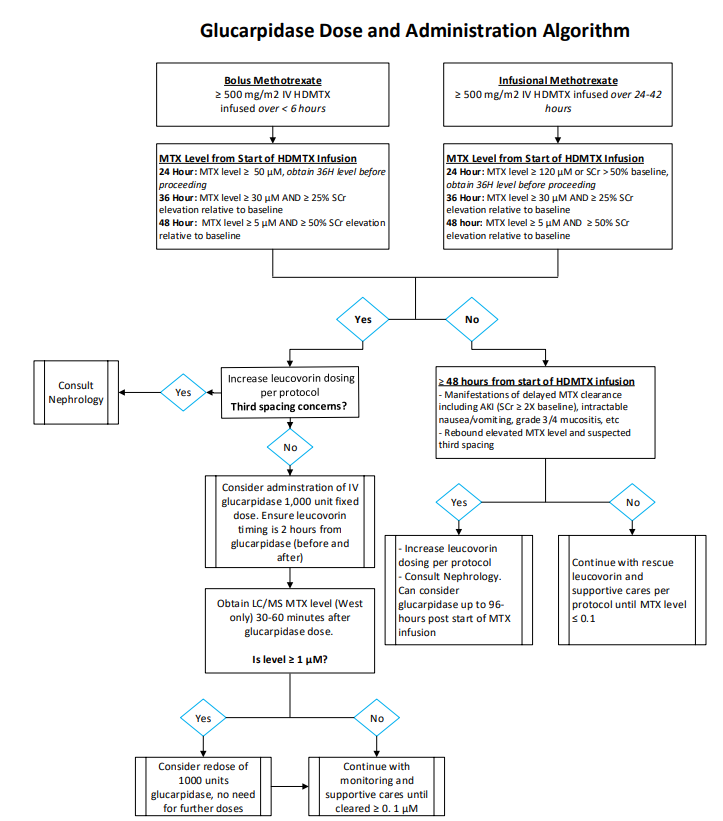

Supplement: Supplementary file 2 — Additional file 2. Institutional algorithm for fixed-dose glucarpidase administration. [file 13256_2025_5774_MOESM2_ESM.docx]
